# Sensory Processing Patterns and Motor Proficiency in Youth Football Players: A Cross-Sectional Study

**DOI:** 10.3390/sports14030118

**Published:** 2026-03-17

**Authors:** Sultan Akel, Çiğdem Öksüz

**Affiliations:** 1 Department of Occupational Therapy, Faculty of Health Sciences, İstanbul Atlas University, İstanbul 34403, Turkey; 2 Department of Occupational Therapy, Faculty of Health Sciences, Hacettepe University, Ankara 06100, Turkey; coksuz@hacettepe.edu.tr

**Keywords:** sensory processing, motor proficiency, balance, sensorimotor integration, youth athletes

## Abstract

Background: Sensory processing and motor proficiency contribute to movement regulation in adolescent athletes. While motor competence has been widely studied in youth football, the role of trait-level sensory processing remains underexplored. This study examined associations between sensory processing patterns and motor proficiency in adolescent football players. Methods: In this cross-sectional study, 116 male youth football players (mean age: 14.16 ± 1.55 years) from a professional academy completed the Adolescent/Adult Sensory Profile and the Bruininks–Oseretsky Test of Motor Proficiency, Second Edition, Brief Form (BOT-2 BF). Spearman correlations were computed across 36 sensory–motor comparisons, with false discovery rate (FDR) correction applied. Partial correlations controlled for age and years of training. Results: After FDR correction, sensation seeking showed a moderate positive association with fine motor precision (ρ = 0.49, *p* < 0.001). Low registration demonstrated a large negative association with fine motor integration (ρ = −0.61, *p* < 0.001) and small-to-moderate negative associations with bilateral coordination and balance (|ρ| = 0.27–0.32). These associations remained significant after adjustment. Conclusions: Sensory processing patterns were differentially associated with coordination- and balance-related motor domains. Findings should be considered exploratory and warrant longitudinal and sport-specific investigation.

## 1. Introduction

Football is a high-intensity team sport that places substantial physical, neuromuscular, and cognitive demands on players, especially during adolescence, when training volume and competitive exposure increase significantly [1]. Youth athletes must adapt continuously to growth-related changes in body proportions, coordination, and postural control when participating in structured training programs. Contemporary sports science increasingly recognizes that motor development during adolescence is shaped not only by training exposure, but also by individual neurobehavioral characteristics that influence movement regulation [2,3].

Motor proficiency, defined as the ability to efficiently and appropriately perform a variety of fine and gross motor tasks, is recognized as a fundamental contributor to football performance. Higher motor proficiency facilitates the acquisition of sport-specific techniques, improves movement efficiency, and enables adaptive responses to complex game situations [3,4]. In youth football, motor proficiency has been linked to superior technical skill performance and improved balance, both of which are important for optimizing performance [5,6]. However, although motor competence has been widely studied, little attention has been given to the sensory processes underlying individual differences in motor coordination and postural stability among typically developing adolescent athletes.

Sensory processing refers to the neurobehavioral mechanisms through which individuals detect, modulate, and respond to sensory input from their bodies and environments. According to Dunn’s sensory processing model, individual differences exist across four quadrants: low registration, sensory sensitivity, sensation avoidance, and sensation seeking. These quadrants reflect variations in neurological thresholds and behavioral self-regulation strategies [7,8]. While sensory processing has been extensively studied in clinical and developmental populations, there is growing evidence that sensory-motor integration and perceptual-motor coupling are also relevant to motor skill execution in athletic contexts [9,10,11].

In dynamic sports environments, effective motor coordination depends on integrating multisensory information, such as visual, vestibular, proprioceptive, and tactile inputs. Studies on perceptual-cognitive expertise in football have shown that visual-motor coordination, anticipatory control, and sensorimotor integration distinguish performance levels between youth and elite players [9,10,12]. However, these studies tend to focus on sport-specific perceptual skills rather than on broader sensory processing characteristics at the trait level. There is a lack of empirical studies directly examining the associations between standardized sensory processing measures and general motor proficiency domains in adolescent football players.

This study investigated the relationship between sensory processing patterns and motor proficiency in adolescent football players. By investigating the relationship between sensory processing characteristics and motor domains related to coordination, balance, and movement efficiency, this study contributes to the growing body of research on sensory-motor factors relevant to the development of young athletes. Additionally, it provides a foundation for future studies that incorporate sport-specific performance measures and longitudinal outcomes.

## 2. Materials and Methods

### 2.1. Study Design

This quantitative, cross-sectional study was designed to explore the relationship between sensory processing patterns and general motor proficiency in adolescent football players.

### 2.2. Participants and Procedure

Participants were recruited from the youth academy of a professional football club, which is characterized by standardized coaching practices, structured competitive exposure, and progressive training loads. This setting provided a consistent training environment suitable for examining sensory-motor characteristics in an organized youth sports context.

Eligible participants were male adolescent athletes between 11 and 17 years of age who were registered with the academy and actively training. The participants represented multiple competitive age categories within the academy structure. Inclusion criteria required the ability to independently complete study assessments and provide written informed assent, as well as parental or legal guardian consent. Athletes were excluded if they had a diagnosed neurological, psychiatric, or developmental disorder that could affect sensory or motor functioning. Playing experience referred to the total number of years participating in organized football, not just academy tenure. Biological maturation status (e.g., peak height velocity) was not assessed. Due to the wide age range, chronological age was treated as a continuous variable and statistically controlled in subsequent analyses to account for maturational variability.

An a priori power analysis determined the minimum required sample size to be 105 (r = 0.30, α = 0.05, power = 0.85). All 213 registered athletes and their parents were provided with study information and invited to participate. Of these athletes, 161 were participating in training sessions at the time of recruitment. Twenty-nine newly registered players were excluded because they did not meet the inclusion criteria, and 13 were excluded due to chronic illness. Three of the remaining 119 eligible athletes did not receive parental permission to participate. The final sample included 116 male adolescent football players.

### 2.3. Measurements

Assessments were conducted individually by trained researchers within the club’s training facility to maintain ecological relevance to the football environment while ensuring standardized testing conditions. Before testing began, participants completed a form documenting their age, playing position, and how long they had been training in organized football.

#### 2.3.1. Bruininks–Oseretsky Test of Motor Proficiency, Second Edition, Brief Form (BOT-2 BF)

Motor proficiency was assessed using the Bruininks-Oseretsky Test of Motor Proficiency, Second Edition, Brief Form (BOT-2 BF) [13]. The BOT-2 BF consists of eight subtests with 12 items total and takes approximately 15–20 min to administer. These subtests evaluate fine motor precision, fine motor integration, manual dexterity, bilateral coordination, balance, speed and agility, upper-limb coordination, and strength. Item point scores were used for all analyses. The BOT-2 manual reports strong internal consistency for the Brief Form (α ≈ 0.87). The validity and reliability of the Turkish version have been established [14]. The BOT-2 Brief Form is designed primarily as a screening instrument. In high-performing youth academy cohorts, however, ceiling effects and restricted score variance may occur, particularly in subtests with a limited scoring range (e.g., upper-limb coordination). Restricted variance may attenuate correlation coefficients, which was considered when interpreting effect sizes.

#### 2.3.2. Adolescent/Adult Sensory Profile (AASP)

Sensory processing patterns were assessed using the Adolescent/Adult Sensory Profile (AASP), a standardized self-report questionnaire based on Dunn’s four-quadrant sensory processing model [7]. The AASP evaluates differences in four sensory processing patterns: low registration, sensory sensitivity, sensation avoidance, and sensation seeking. The AASP was used to assess sensory responsiveness. The AASP evaluates generalized patterns of sensory responsiveness reflecting stable individual differences in daily contexts. The validity and reliability of the Turkish version of the AASP have previously been demonstrated [15]. Questionnaire completion was supervised by a trained researcher to ensure understanding of item content and standardized administration conditions. All questionnaires were reviewed upon completion. No missing data were observed; therefore, no imputation procedures were required.

### 2.4. Data Analysis

Analyses were conducted using IBM SPSS Statistics (version 25). Descriptive statistics (mean, standard deviation, minimum, and maximum) were calculated for AASP quadrant scores and BOT-2 BF subtest and total point scores. Normality was assessed using the Shapiro–Wilk test. As most variables deviated from normality, nonparametric procedures were applied. Spearman’s rank-order correlations were used to examine associations between the four AASP quadrants and nine BOT-2 BF outcomes (eight subtests and the total score). To control for multiple comparisons across the 36 sensory–motor correlation tests (4 AASP quadrants × 9 BOT-2 outcomes), the Benjamini–Hochberg false discovery rate procedure (FDR, q = 0.05) was applied. Because raw BOT-2 point scores were used, partial Spearman correlations were computed using rank-transformed variables while controlling for chronological age and years of organized football participation. Correlation magnitudes were interpreted as small (|r| = 0.10–0.29), moderate (|r| = 0.30–0.49), or large (|r| ≥ 0.50). Group differences by playing position were examined using Kruskal–Wallis H tests, with epsilon-squared reported as a measure of nonparametric effect size. An additional exploratory subgroup analysis was conducted to examine potential developmental variation. Participants were divided into early adolescence (11–13 years, *n* = 57) and mid-adolescence (14–17 years, *n* = 59). Spearman correlations between low registration and coordination-related motor domains were computed separately within each age group. These subgroup analyses were considered exploratory and were not adjusted for multiple comparisons. An a priori power analysis indicated that a minimum sample size of 105 participants would be required to detect a moderate correlation (r = 0.30) with α = 0.05 and power = 0.85. The achieved sample size (*N* = 116) exceeded this requirement, indicating sufficient statistical power for the primary analyses.

### 2.5. Ethical Approval

This study was conducted in accordance with the principles of the Declaration of Helsinki. Ethical approval was obtained from the İstanbul Atlas University Ethics Committee (Approval No: E-22686390-050.99-72692, Approval date: 28 July 2025). All participants and their parents were informed about the purpose and procedures of the study, and written informed consent was obtained prior to participation.

## 3. Results

### 3.1. Demographic Characteristics

The sample included 116 male adolescent football players with an average age of 14.16 ± 1.55 years. Their playing experience ranged from one to four years (mean = 2.59 ± 0.89 years), reflecting a youth academy cohort exposed to structured training loads and regular competitive demands. Of the 116 players, 46 were forwards (39.7%), 46 were defenders (39.7%), 18 were midfielders (15.5%), and 6 were goalkeepers (5.2%).

The distribution of sensory processing scores across the four quadrants is presented in Table 1. For all quadrants, the majority of participants scored within the typical range (similar to most people). Smaller proportions were classified as having scores less or much more than most people, with only a few participants falling into the extreme categories (much less or much more).

Table 2 presents descriptive statistics for the BOT-2 BF subtest and total scores. These scores indicate a relatively homogeneous motor proficiency profile across the sample, reflecting comparable technical and movement-related capacities within this youth academy cohort.

### 3.2. Correlation Analysis

Following Spearman correlation analyses and the application of the Benjamini–Hochberg false discovery rate correction (q = 0.05), several sensory-motor associations remained statistically significant (Table 3). Sensation seeking demonstrated a moderate-to-large positive association with fine motor precision (subtest 1; r = 0.487, *p* < 0.001). Low registration showed a large negative association with fine motor integration (subtest 2; r = −0.610, *p* < 0.001) and small-to-moderate negative associations with bilateral coordination (subtest 4; r = −0.316, *p* = 0.010) and balance (subtest 5; r = −0.266, *p* = 0.004). A small-to-moderate positive association was observed between low registration and manual dexterity (subtest 3; r = 0.297, *p* = 0.001). Sensory sensitivity demonstrated a small-to-moderate negative association with balance (subtest 5; r = −0.278, *p* = 0.003). As expected, strong positive associations were observed between speed and agility (subtest 6) and the total BOT-2 score (r = 0.617, *p* < 0.001), as well as between strength (subtest 8) and the total score (r = 0.650, *p* < 0.001). These results reflect the contribution of these subtests to overall motor proficiency. Other tested associations did not remain significant following FDR correction. A complete correlation matrix including all tested associations, corresponding *p*-values, and FDR-adjusted results is provided in Supplementary Table S1.

A Kruskal–Wallis H test was conducted to investigate differences in AASP quadrant scores and BOT-2 BF subtest and total scores across playing positions. No statistically significant differences were found for sensation avoiding (H = 6.16, *p* = 0.104, ε^2^ = 0.03), sensation seeking (H = 3.64, *p* = 0.303, ε^2^ = 0.01), low registration (H = 1.32, *p* = 0.726, ε^2^ = 0.00), or sensory sensitivity (H = 4.95, *p* = 0.176, ε^2^ = 0.02) across positions. Similarly, no significant differences were observed across positions for BOT-2 BF subtest scores (e.g., Subtest 1: H = 2.57, *p* = 0.463, ε^2^ = 0.00) or the total score (*p* > 0.05).

After controlling for chronological age and years of football participation, the correlation between sensation seeking and fine motor precision remained moderate (r = 0.46, *p* < 0.001). The negative association between low registration and fine motor integration remained substantial (r = −0.58, *p* < 0.001), while the associations with bilateral coordination and balance remained small to moderate (rs ranging from −0.25 to −0.30, *p* < 0.05). The previously observed association between low registration and manual dexterity was no longer statistically significant after adjustment (*p* > 0.05). Sensory sensitivity remained negatively associated with balance (r = −0.26, *p* = 0.004).

Exploratory subgroup analyses revealed that the negative associations between low registration and coordination-related motor domains varied by age group. Among early adolescents (ages 11–13), low registration was negatively associated with fine motor integration (r = −0.30, *p* = 0.025) and bilateral coordination (r = −0.42, *p* = 0.001), and a trend-level association was observed for balance (r = −0.25, *p* = 0.067). In contrast, in the mid-adolescent group (ages 14–17), the associations between low registration and fine motor integration (r = 0.13, *p* = 0.317) and bilateral coordination (r = 0.20, *p* = 0.125) were not statistically significant. However, a small negative association persisted between low registration and balance (r = −0.28, *p* = 0.031).

## 4. Discussion

This study examined the relationship between sensory processing patterns and general motor proficiency in adolescent football players. The findings suggest that sensory characteristics at the trait level are associated with specific motor domains, particularly those involving coordination and balance. These results add to the growing body of literature emphasizing the multidimensional nature of motor development during adolescence [2,11].

The moderate observed association between sensation seeking and fine motor precision suggests that individual differences in sensory responsiveness may be related to how adolescents engage with tasks that require precise motor control. Sensation seeking has been described as reflecting lower neurological thresholds and active self-regulation strategies [7], both of which may influence interaction with motor tasks. However, due to the cross-sectional design of this study, these findings should be interpreted as correlational rather than as evidence of causal mechanisms.

The strong negative correlation between low registration and fine motor integration, as well as the weaker correlations with bilateral coordination and balance, suggests that higher sensory thresholds may be related to variability in specific, coordination-related motor domains. Balance and postural control are important components of motor competence during adolescence [16,17]. The present findings extend this literature by suggesting that sensory processing patterns at the trait level may be associated with individual differences in these domains within a typically developing athletic cohort.

Importantly, these associations remained significant after statistically controlling for chronological age and years of football participation, although exploratory subgroup analyses indicated developmental variation in their strength. This suggests that they are not solely attributable to developmental stage or training exposure. However, biological maturation was not directly assessed. Future research that incorporates maturation indicators (e.g., peak height velocity) would provide a more comprehensive understanding of these relationships. Notably, the association between low registration and manual dexterity was no longer significant after partial adjustment. This suggests that the initially observed relationship may have been influenced by age- or training-related factors rather than reflecting a stable sensory-motor linkage. This finding highlights the importance of considering maturational and experiential variables when interpreting sensory-motor associations in adolescent populations.

The absence of positional differences suggests that sensory processing characteristics and general motor proficiency may represent broader developmental attributes rather than adaptations specific to a playing position during early and mid-adolescence. This finding aligns with existing literature indicating that pronounced positional specialization often emerges later in development [18,19]. Effect size estimates for positional comparisons were small (ε^2^ ≤ 0.03), further supporting the absence of meaningful positional effects. However, the goalkeeper subgroup was small (n = 6), which may have limited statistical power to detect subtle subgroup differences. Therefore, the absence of significant findings across playing positions should be interpreted cautiously, particularly for less represented positions.

Notably, exploratory subgroup analyses indicated that these associations were stronger in early adolescence (ages 11–13) and weaker in mid-adolescence (ages 14–17). This pattern may reflect developmental changes in sensory integration and motor coordination during adolescence. During this period, neuromuscular maturation and compensatory strategies may reduce the observable impact of sensory processing variability [20]. However, due to the exploratory nature of these analyses and the reduced statistical power within subgroups, these findings should be interpreted with caution and confirmed in longitudinal studies.

These findings provide preliminary insight into sensory-motor associations in youth football. However, several considerations are warranted. Using BOT-2 point scores and observing limited variability in certain subtests (e.g., upper-limb coordination) may have reduced detectable associations. Additionally, the study did not evaluate sport-specific performance metrics, training adaptations, or injury outcomes. Longitudinal research that incorporates field-based performance measures would clarify the prospective association between sensory processing characteristics and sport-related outcomes.

Additionally, an important methodological consideration is ecological validity. Sensory processing was assessed using a self-report instrument that reflects generalized sensory responsiveness in daily contexts. Motor proficiency, on the other hand, was evaluated using a standardized clinical assessment. While these tools provide reliable, controlled measurements, they may not fully capture the dynamic perceptual-motor demands of football performance in competitive environments. Future studies that incorporate sport-specific perceptual-motor tasks and field-based assessments may provide greater ecological validity.

Overall, this exploratory study highlights that individual differences in generalized sensory processing patterns are associated with specific domains of motor proficiency in adolescent football players. These results emphasize the importance of considering sensory-motor variability in youth sports research while recognizing the need for longitudinal and sport-specific investigations.

### Limitations and Future Directions

Several limitations should be acknowledged. First, the cross-sectional design precludes drawing causal inferences about the associations between sensory processing and motor proficiency. Longitudinal studies are necessary to determine the temporal direction and developmental stability of these relationships. Second, while chronological age was statistically controlled for, biological maturation status (e.g., peak height velocity) was not directly assessed. Due to the wide age range (11–18 years), maturational variability may have influenced the outcomes of motor performance. Third, BOT-2 Brief Form point scores were used instead of age-normed standard scores. While age was controlled analytically, using non-normed scores may have introduced residual age-related variance. Additionally, since the BOT-2 Brief Form is primarily a screening instrument, restricted score variability and potential ceiling effects, particularly regarding upper-limb coordination, may have reduced the magnitude of detectable associations. Fourth, the study only included male adolescent athletes, which limits its generalizability to female football populations and other youth sports contexts. Finally, the study did not include sport-specific performance or injury-related measures. More longitudinal research incorporating field-based assessments is needed to determine the prospective association between sensory processing characteristics and sport-related outcomes.

## 5. Conclusions

This exploratory study identified statistically significant associations between generalized sensory processing patterns and specific motor proficiency domains in adolescent football players. Within this cohort, sensory processing traits were differentially related to coordination- and balance-related motor domains. These findings should be interpreted as associative rather than causal, reflecting relationships observed under controlled assessment conditions. Due to the cross-sectional design and absence of sport-specific performance measures, the results offer preliminary insight rather than concrete evidence of practical applications. Future research employing longitudinal designs and sport-specific perceptual-motor assessments is needed to clarify how sensory processing characteristics relate to football-specific performance.

## Figures and Tables

**Table 1:** Descriptive Statistics and Normative Distribution of AASP Quadrant Scores (Age 11–17).

Quadrant	Min–Max	Mean ± SD	Much Less than Most People n (%)	Less than Most People n (%)	Similar to Most People n (%)	More than Most People n (%)	Much More than Most People n (%)
Low Registration	21–47	33.27 ± 7.29	6 (5.2)	28 (24.1)	70 (60.4)	10 (8.6)	2 (1.7)
Sensation Seeking	27–62	44.66 ± 7.14	3 (2.6)	15 (12.9)	78 (67.2)	16 (13.8)	4 (3.5)
Sensory Sensitivity	20–56	38.15 ± 8.66	4 (3.5)	18 (15.5)	68 (58.6)	20 (17.2)	6 (5.2)
Sensation Avoiding	21–55	37.84 ± 8.08	2 (1.7)	22 (19.0)	72 (62.1)	16 (13.8)	2 (1.7)

AASP: Adolescent/Adult Sensory Profile.

**Table 2:** Descriptive Statistics of BOT-2 BF Subtests and Total Scores.

BOT-2 Subtests	Min–Max	Mean ± SD
Subtest 1-Fine Motor Precision	1–4	2.44 ± 0.69
Subtest 2-Fine Motor Integration	2–5	4.61 ± 0.86
Subtest 3-Manual Dexterity	1–6	3.22 ± 1.03
Subtest 4-Bilateral Coordination	2–3	2.68 ± 0.47
Subtest 5-Balance	1–4	3.66 ± 0.73
Subtest 6-Speed and Agility	0–10	6.15 ± 2.93
Subtest 7-Upper-Limb Coordination	5–6	5.96 ± 0.19
Subtest 8-Strength	3–9	8.30 ± 1.55
Total Score	37–63	52.67 ± 5.56

BOT-2 BF: Bruininks-Oseretsky Test of Motor Proficiency 2 Brief Form.

**Table 3:** Significant Correlations Between AASP Quadrants and BOT-2 BF Scores.

Variable 1	Variable 2	r	*p*-Value
Sensation Seeking	Subtest 1	0.357	<0.001
Low Registration	Subtest 2	−0.610	<0.001
Low Registration	Subtest 3	0.297	0.001
Low Registration	Subtest 4	−0.316	0.010
Low Registration	Subtest 5	−0.266	0.004
Sensory Sensitivity	Subtest 5	−0.278	0.003
BOT-2 Subtest 6	Total Score	0.617	<0.001
BOT-2 Subtest 8	Total Score	0.650	<0.001

AASP: Adolescent/Adult Sensory Profile. BOT-2 BF: Bruininks-Oseretsky Test of Motor Proficiency 2 Brief Form. Exact *p*-values are reported to three decimal places; values below 0.001 are reported as *p* < 0.001.

## Data Availability

The data presented in this study are not publicly available due to ethical restrictions and the inclusion of identifiable information related to youth athletes.

## Supplementary Materials

The following supporting information can be downloaded at: https://www.mdpi.com/article/10.3390/sports14030118/s1. Supplementary Table S1: Spearman Correlation Matrix Between Sensory Processing Quadrants (AASP) and BOT-2 Brief Form Outcomes (*N* = 116).
