# Supplementary material for: Sensory Processing Patterns and Motor Proficiency in Youth Football Players: A Cross-Sectional Study
Source: Sports (Basel). 2026 Mar 17;14(3):118. doi: 10.3390/sports14030118 (PMC13029813; doi:10.3390/sports14030118)
Supplement: Supplementary file 1 [file sports-14-00118-s001.zip › sports-4146370-supplementary.pdf]

Table S1: Spearman Correlation Matrix Between Sensory Processing Quadrants (AASP) and BOT-2 Brief Form Outcomes (N = 116)

**Variable 1**	**Variable 2**	**r**	**p-value**	**FDR-adjusted p-value**	**Significant after FDR correction (q = .05)**
Sensation Avoidance	Fine Motor Precision	-0.046	0.626	0.693	FALSE
Sensation Avoidance	Fine Motor Integration	-0.114	0.221	0.472	FALSE
Sensation Avoidance	Manual Dexterity	0.071	0.449	0.627	FALSE
Sensation Avoidance	Bilateral Coordination	-0.094	0.316	0.542	FALSE
Sensation Avoidance	Balance	-0.158	0.091	0.272	FALSE
Sensation Avoidance	Speed and Agility	0.059	0.529	0.677	FALSE
Sensation Avoidance	Upper-Limb Coordination	-0.112	0.231	0.472	FALSE
Sensation Avoidance	Strength	-0.044	0.639	0.693	FALSE
Sensation Avoidance	Total Score	-0.114	0.224	0.472	FALSE
Sensation Seeking	Fine Motor Precision	0.357	0.000	0.000	TRUE
Sensation Seeking	Fine Motor Integration	0.331	0.452	0.005	FALSE
Sensation Seeking	Manual Dexterity	-0.073	0.434	0.627	FALSE
Sensation Seeking	Bilateral Coordination	0.064	0.497	0.662	FALSE
Sensation Seeking	Balance	0.109	0.243	0.472	FALSE
Sensation Seeking	Speed and Agility	-0.234	0.411	0.445	FALSE
Sensation Seeking	Upper-Limb Coordination	0.073	0.439	0.627	FALSE
Sensation Seeking	Strength	-0.099	0.29	0.522	FALSE
Sensation Seeking	Total Score	0.057	0.546	0.677	FALSE
Low Registration	Fine Motor Precision	-0.247	0.456	0.134	FALSE
Low Registration	Fine Motor Integration	-0.610	0.000	0.000	TRUE
Low Registration	Manual Dexterity	0.297	0.001	0.015	TRUE
Low Registration	Bilateral Coordination	-0.316	0.010	0.011	TRUE

Low Registration	Balance	-0.266	0.004	0.023	TRUE
Low Registration	Speed and Agility	0.108	0.249	0.472	FALSE
Low Registration	Upper-Limb Coordination	-0.217	0.019	0.07	FALSE
Low Registration	Strength	-0.07	0.453	0.627	FALSE
Low Registration	Total Score	-0.201	0.031	0.101	FALSE
Sensory Sensitivity	Fine Motor Precision	-0.137	0.142	0.395	FALSE
Sensory Sensitivity	Fine Motor Integration	-0.09	0.338	0.553	FALSE
Sensory Sensitivity	Manual Dexterity	0.042	0.654	0.693	FALSE
Sensory Sensitivity	Bilateral Coordination	0.006	0.951	0.951	FALSE
Sensory Sensitivity	Balance	-0.278	0.003	0.018	TRUE
Sensory Sensitivity	Speed and Agility	0.015	0.874	0.899	FALSE
Sensory Sensitivity	Upper-Limb Coordination	-0.044	0.637	0.693	FALSE
Sensory Sensitivity	Strength	-0.044	0.636	0.693	FALSE

AASP:Adolescent/Adult Sensory Profile
BOT-2 BF: Bruininks-Oseretsky Test of Motor Proficiency 2 Brief Form
